# Supplementary figures and images for: Mitigating NaCl stress in Vigna radiata L. cultivars using Bacillus pseudomycoides
Source: PeerJ. 2024 Jun 4;12:e17465. doi: 10.7717/peerj.17465 (PMC11160433; doi:10.7717/peerj.17465)

Inqelab mung (V1)

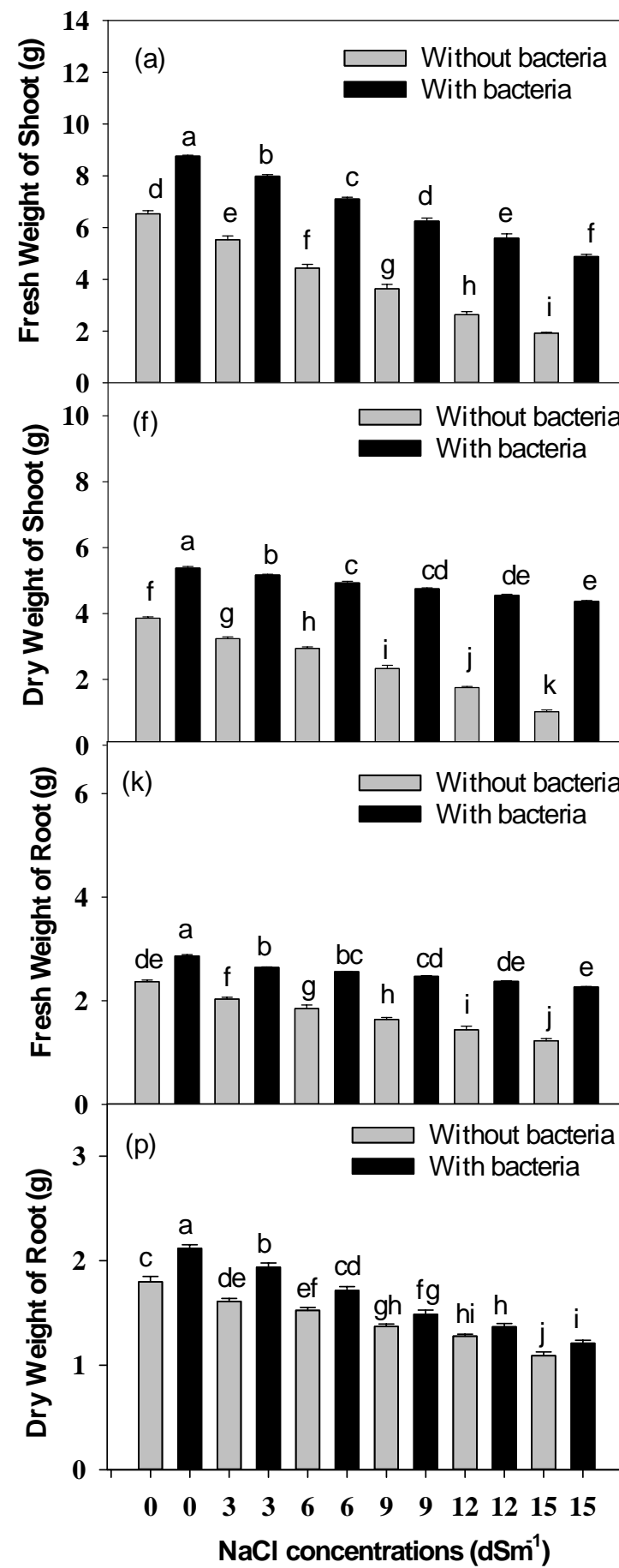

NIFA-19 (V2)

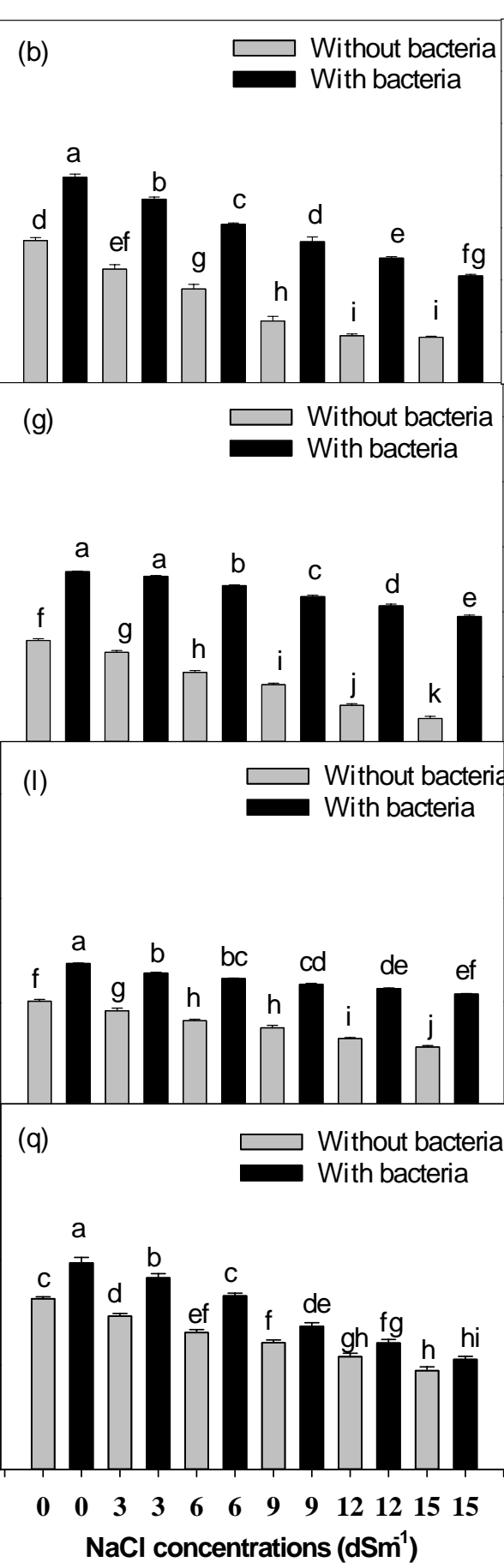

NIFA-17(V3)

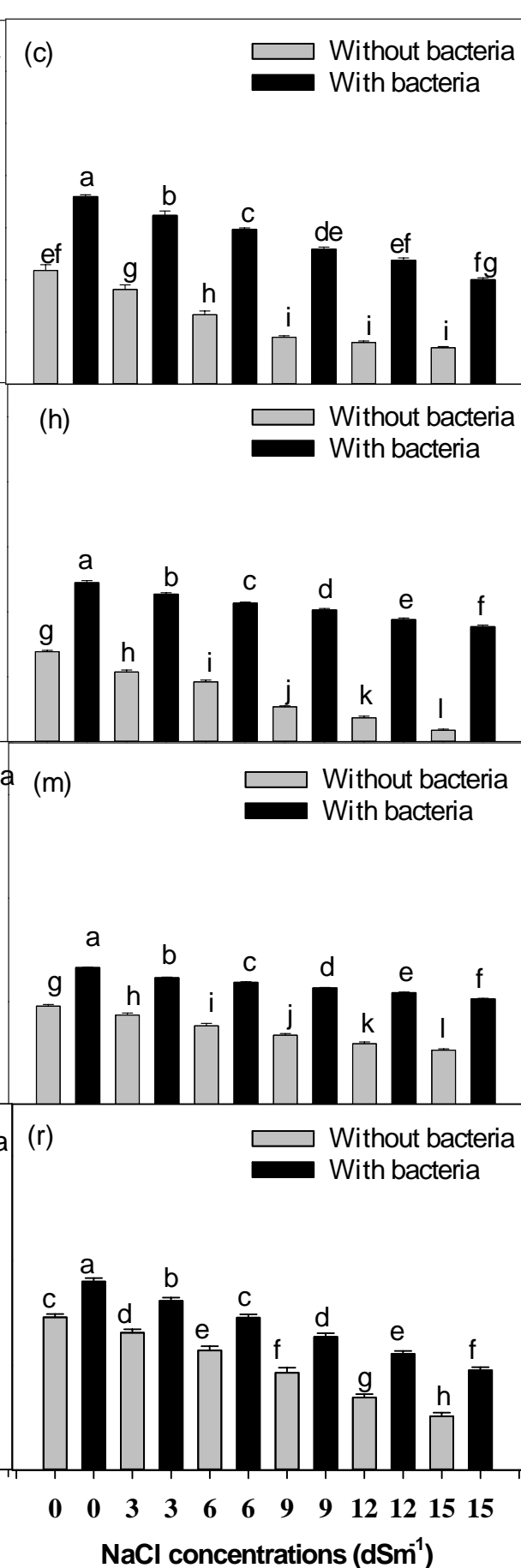

Sona mung (V4)

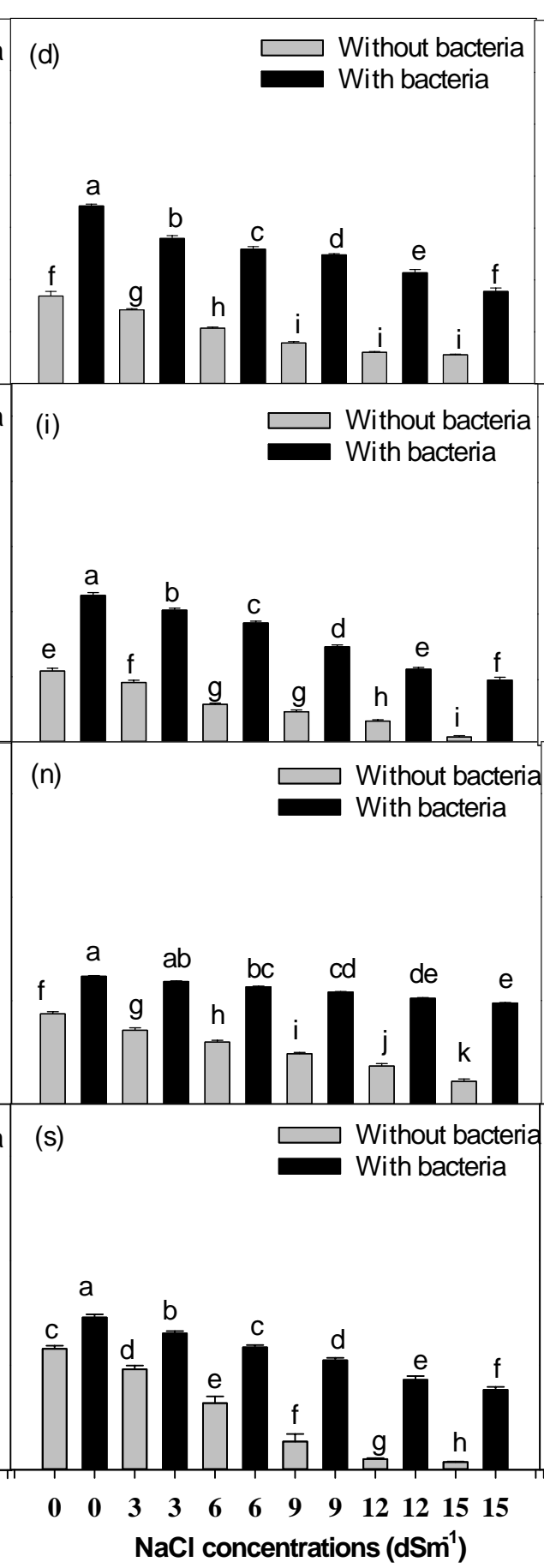

Ramzan mung (V5)

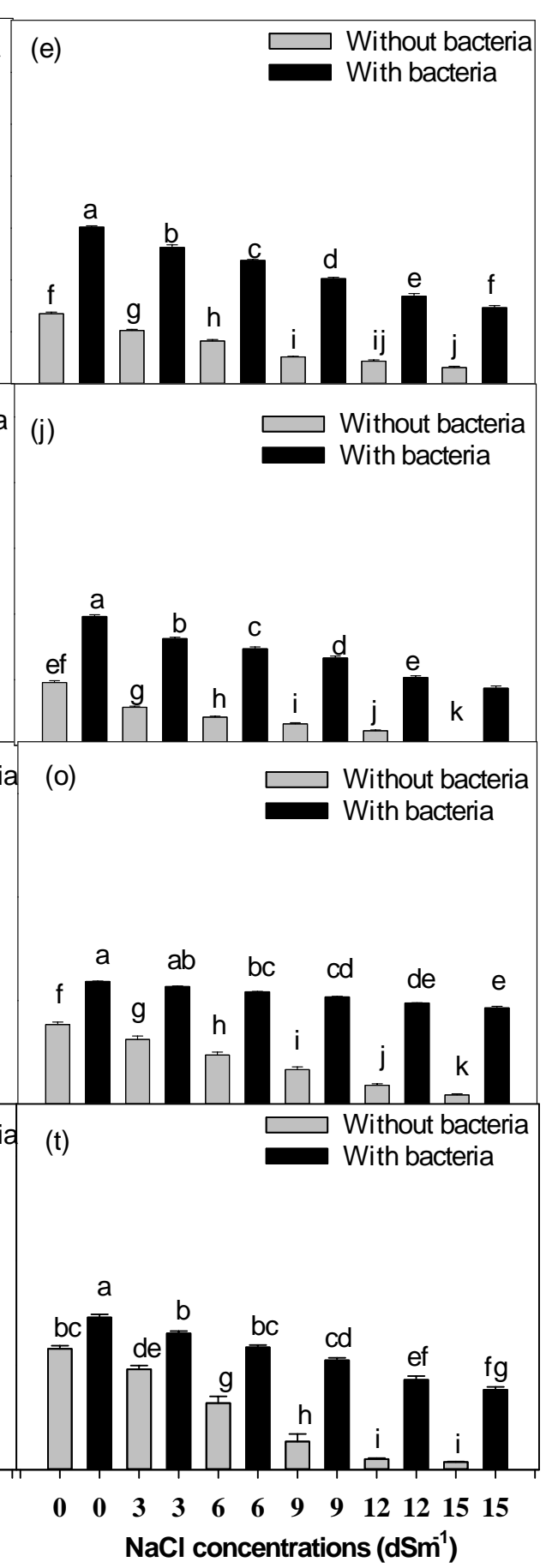

Supplement: Supplemental Information 1 — Bars indicate mean (±) standard error and different letters mean significant differences. Grey bars indicate soil with NaCl treatment while black bars indicate NaCl treatment combined with B. pseudomycoides inoculum. [file peerj-12-17465-s001.pdf]

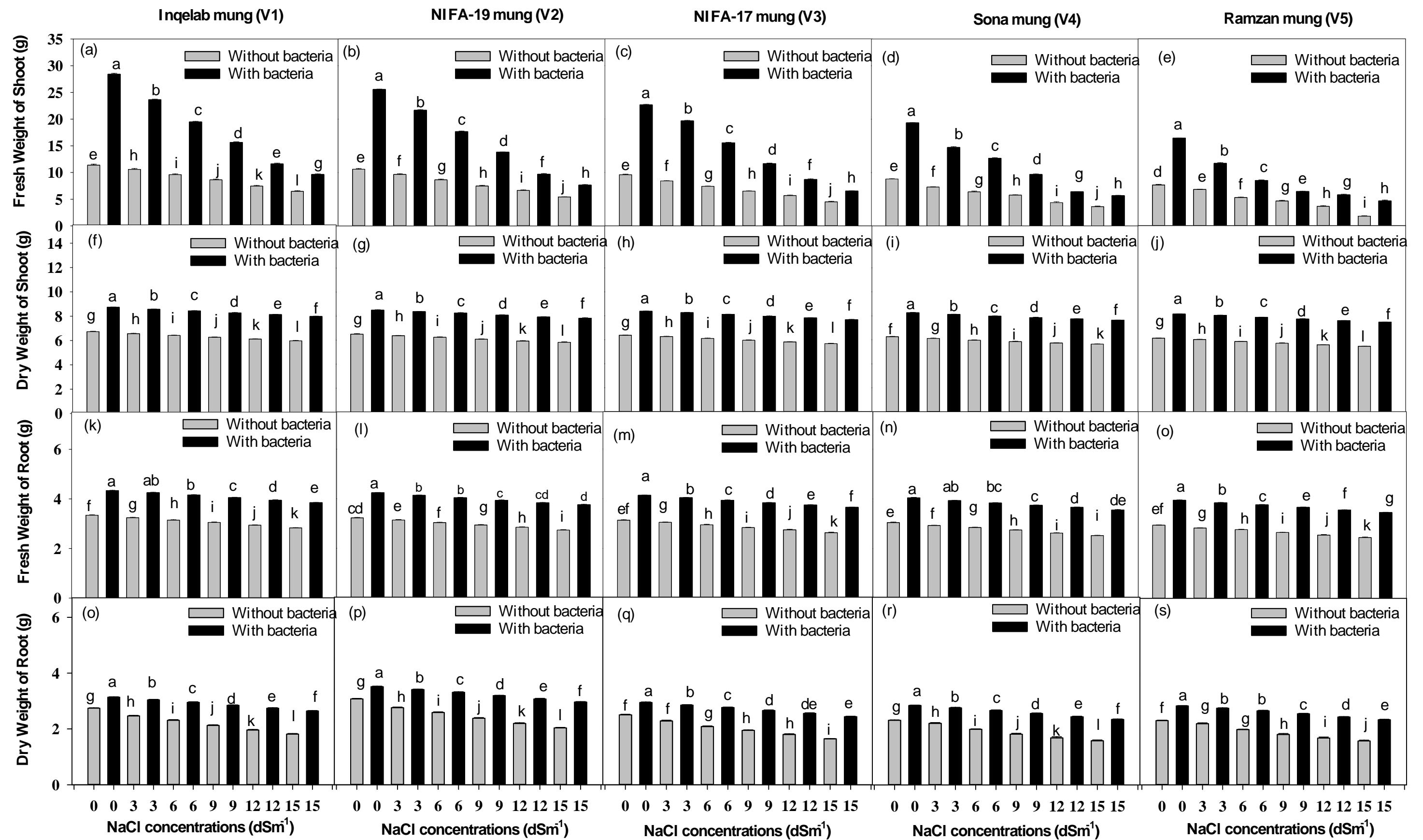

Supplement: Supplemental Information 2 — Bars indicate mean (±) standard error and different letter (s) mean significant differences. Grey bars indicate soil with NaCl treatment while black bars indicate NaCl treatment combined with B. pseudomycoides inoculum. [file peerj-12-17465-s002.pdf]

Inqelab mung (V1)

NIFA-19 (V2)

NIFA-17 (V3)

Sona mung (V4)

Ramzan mung (V5)

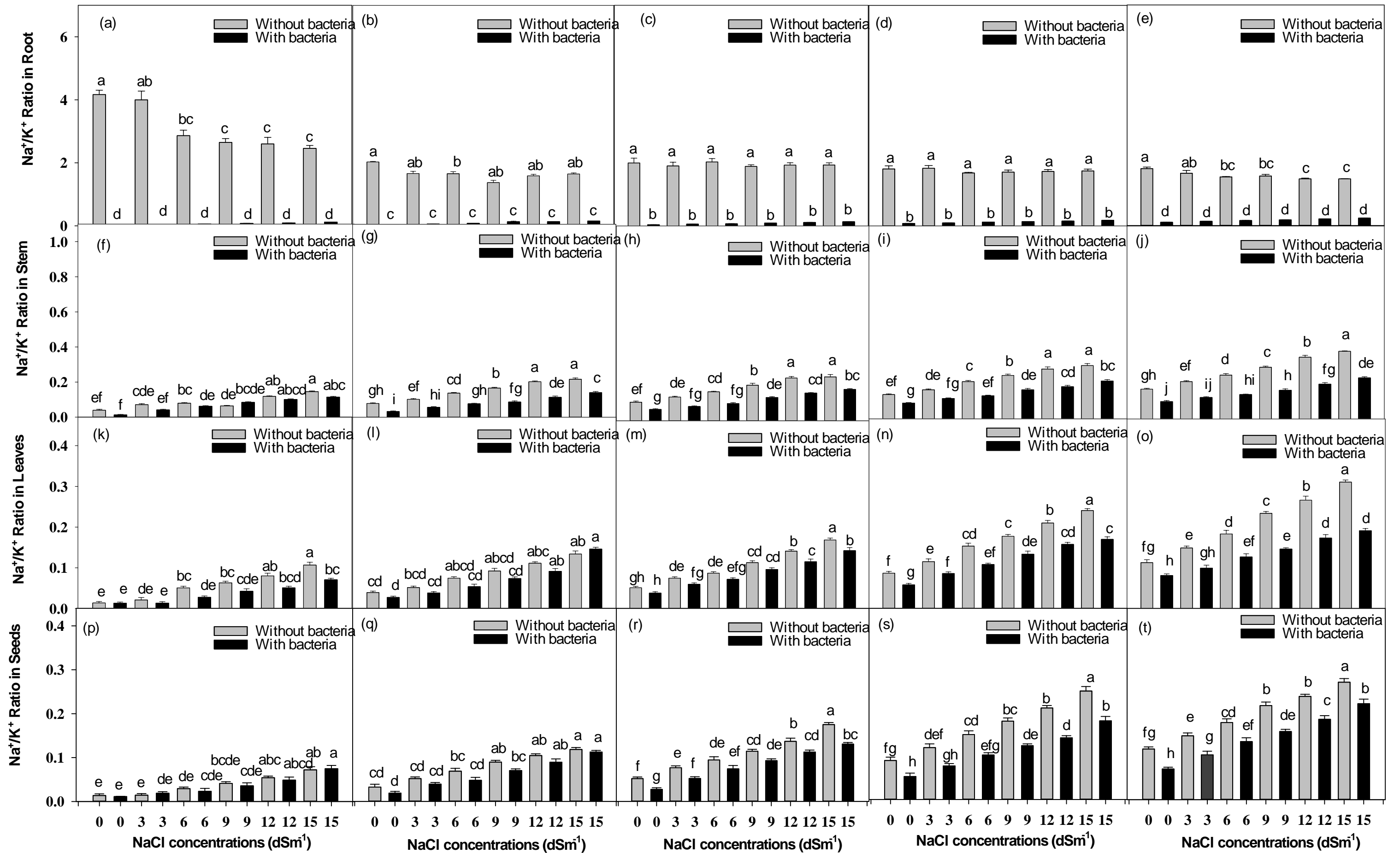

Supplement: Supplemental Information 3 — Bars indicate mean (±) standard error and different letter (s) mean significant differences. Grey bars indicate soil with NaCl treatment, while black bars indicate NaCl treatment combined with B. pseudomycoides inoculum. [file peerj-12-17465-s003.pdf]
